# From the Editor's Desk

**Published:** 2014

**Authors:** PJ Commerford

**Affiliations:** Editor-in-Chief

This issue contains useful information on patterns of disease presentation in Africa. Grimaldi and colleagues (page 204) document the pattern of structural heart disease causing heart failure in patients presenting to a tertiary hospital in Kampala. Many were young and suffered from rheumatic heart disease (RHD) and congenital heart disease (CHD). One suspects this was a highly selected group as the patients were identified during NGO missions, presumably aimed at identifying suitable candidates for surgery. Nonetheless the article reflects the importance of RHD as a cause of disability and death in the young in Uganda, as in many other parts of Africa, and emphasises the need for efforts to improve primary and secondary prevention of this eminently preventable disease.

Another aspect of the heart failure spectrum is presented by Ogah and colleagues (page 217). A registry of patients admitted to hospital with acute heart failure and followed for six months showed a pattern of disease different from that found in high-income countries. Patients were younger, the aetiology of the heart failure was most commonly hypertensive heart disease, and an ischaemic aetiology was uncommon. In another article investigating hypertensive heart disease, Ojji and colleagues (page 233) report that brain natriuretic peptide is useful in evaluating cardiac remodelling in African patients with hypertension.

Thrombolysis for acute ischaemic stroke is, I suspect, used much less frequently in Africa than in many other parts of the world because of resource constraints. It is helpful to learn from von Klemperer and co-workers (page 224) that predictors for the serious complication of intracranial haemorrhage, developed elsewhere, apply in an African setting, although it is important to recognise that the demographics of the population of Cape Town differ considerably from the rest of Africa.

